# Identifying pre-conception and pre-natal periods in which ambient air pollution exposure affects fetal growth in the predominately Hispanic MADRES cohort

**DOI:** 10.1186/s12940-022-00925-0

**Published:** 2022-11-26

**Authors:** Alicia K. Peterson, Rima Habre, Zhongzheng Niu, Monica Amin, Tingyu Yang, Sandrah P. Eckel, Shohreh F. Farzan, Fred Lurmann, Nathan Pavlovic, Brendan H. Grubbs, Daphne Walker, Laila A. Al-Marayati, Edward Grant, Deborah Lerner, Theresa M. Bastain, Carrie V. Breton

**Affiliations:** 1 Department of Population and Public Health Sciences, Keck School of Medicine, University of Southern California, Los Angeles, CA 90032 USA; 2 Sonoma Technology Inc., Petaluma, CA 94954 USA; 3 Department of Obstetrics and Gynecology, Keck School of Medicine, University of Southern California, Los Angeles, CA 90033 USA; 4 Department of Radiology, Keck School of Medicine, University of Southern California, Los Angeles, CA 90033 USA; 5 Eisner Health Medical Center, Los Angeles, CA 90015 USA

**Keywords:** Air Pollution, Fetal Growth, Pregnancy, DLM, Health Disparities, Particulate Matter

## Abstract

**Background:**

It is well documented that persons of color experience disproportionate exposure to environmental contaminants, including air pollution, and have poorer pregnancy outcomes. This study assessed the critical windows of exposure to ambient air pollution on in utero fetal growth among structurally marginalized populations in urban Los Angeles.

**Methods:**

Participants (*N* = 281) from the larger ongoing MADRES pregnancy cohort study were included in this analysis. Fetal growth outcomes were measured on average at 32 $\pm$ 2 weeks of gestation by a certified sonographer and included estimated fetal weight, abdominal circumference, head circumference, biparietal diameter and femur length. Daily ambient air pollutant concentrations were estimated for four pollutants (particulate matter less than 2.5 µm (PM_2.5_) and less than 10 µm (PM_10_) in aerodynamic diameter, nitrogen dioxide (NO_2_), and 8-h maximum ozone (O_3_)) at participant residences using inverse-distance squared spatial interpolation from ambient monitoring data. Weekly gestational averages were calculated from 12 weeks prior to conception to 32 weeks of gestation (44 total weeks), and their associations with growth outcomes were modeled using adjusted distributed lag models (DLMs).

**Results:**

Participants were on average 29 years $\pm$ 6 old and predominately Hispanic (82%). We identified a significant sensitive window of PM_2.5_ exposure (per IQR increase of 6 $\mathrm{\mu g}/\mathrm{m}$^3^) between gestational weeks 4–16 for lower estimated fetal weight $\beta$_averaged4-16_ = -8.7 g; 95% CI -16.7, -0.8). Exposure to PM_2.5_ during gestational weeks 1–23 was also significantly associated with smaller fetal abdominal circumference ($\beta$_averaged1-23_ = -0.6 mm; 95% CI -1.1, -0.2). Additionally, prenatal exposure to PM_10_ (per IQR increase of 13 $\mathrm{\mu g}/\mathrm{m}$^3^) between weeks 6–15 of pregnancy was significantly associated with smaller fetal abdominal circumference ($\beta$_averaged6-15 =_ -0.4 mm; 95% CI -0.8, -0.1).

**Discussion:**

These results suggest that exposure to particulate matter in early to mid-pregnancy, but not preconception or late pregnancy, may have critical implications on fetal growth.

**Supplementary Information:**

The online version contains supplementary material available at 10.1186/s12940-022-00925-0.

## Background

Ambient air pollution consists of a diverse mixture of gases and particles and has the ability to potentially affect every organ within the human body [1]. Air pollutants are known contributors to cardiovascular disease and premature mortality with an estimated 135 million Americans currently affected by unhealthy levels of ozone and particulate matter (PM) [2–5]. Additionally, air pollution exposure has been shown to differ dramatically based on race and ethnicity within the United States. A recent study found that exposure to PM with an aerodynamic diameter of 2.5 µm ($\upmu$ m) or less (PM_2.5_), is greater for people of color even after accounting for state, geographic area (urban vs. rural) and income level [6]. Persons from racial and ethnic minority groups experience disproportionate exposures to toxic air emissions due to residential proximity to industrial practices [7, 8] and are more likely to live near traffic sources compared to non-Hispanic whites [9].

In addition to experiencing higher exposures to air pollution, communities of color also tend to have higher rates of adverse birth outcomes compared to non-Hispanic white populations [10]. A myriad of complex factors contribute to these health disparities including racial discrimination, less access to prenatal care, and lower socio-economic status [11, 12]. Another driving factor may be higher levels of exposure to air pollution during pregnancy. Substantial evidence documents that exposure to ambient air pollution during pregnancy influences negative birth outcomes including low birth weight (< 2,500 g) and preterm birth (< 37 weeks) [13–20] due primarily to biological mechanisms impacting the placenta [21–24]. Adverse negative birth outcomes, and decreased fetal growth, influences health across the life course [25–27].

Fetal growth in utero is commonly assessed by sonographic measurements of the fetus’s head circumference, biparietal diameter, abdominal circumference, femur length and estimated fetal weight. Although there is extensive literature on the influence of air pollution on birth outcomes and infant birth weight, there are far fewer studies that have assessed fetal growth and development and the majority of these studies are conducted in more affluent populations. An observational study in Spain found that nitrogen dioxide (NO_2_) exposure during pregnancy was associated with decreased biparietal diameter, abdominal circumference, and estimated fetal weight [28–30]. NO_2_ also has been associated with significant inverse effects on fetal growth in observational cohorts in Los Angeles, California [31], China [32], and the Netherlands [33]. Additionally, studies have shown PM with an aerodynamic diameter of 10 $\upmu$ m or less (PM_10_) [31, 33–37] and PM_2.5_ [34, 38–40] have adverse effects on fetal growth. Observational studies investigating ground level ozone (O_3_) are limited [31, 35] with only one finding significant inverse effects on fetal abdominal circumference [35].

While the literature suggests air pollution adversely impacts in utero fetal growth, direct comparison of results across studies is challenging due to differences in growth outcomes measured, timing of measurements, and differences in exposure assessment and modeling approaches. None of the existing studies addressed finer time resolution of exposure (i.e. weekly) that may be critical for identifying subtle, but important, in utero growth effects. Averaging exposure across pregnancy lacks the ability to identify critical windows of exposure or capture the true variability of the exposures across gestation. Exposure time windows have also generally been limited to prenatal time points, with few studies exploring whether preconception exposures influence fetal growth, which has been shown to influence other pregnancy outcomes [41–43].

To address these gaps, this study assessed the influence of preconception and in utero exposure to four criteria air pollutants including PM_2.5_, PM_10_, NO_2_, and (8-h maximum) O_3_ on third trimester fetal growth within a structurally marginalized population of predominately Hispanic participants. We hypothesized that there are important windows of susceptibility to ambient air pollution exposure that are associated with reduced fetal weight, abdominal circumference, femur length, biparietal diameter and head circumference. We explored this using distributed lag models (DLMs) of exposures spanning from 12 weeks preconception to 32 weeks of gestation. As a secondary aim, we also assessed prenatal air pollution on in utero fetal growth using standard pregnancy-average pollution exposure effects within this population.

## Methods

### Sample

Participants were drawn from the larger ongoing Maternal And Developmental Risks from Environmental and Social Stressors (MADRES) pregnancy cohort study. An overview of the study design, protocol, and demographics of the cohort have been previously described [44]. In brief, participants were enrolled into the study during pregnancy from four prenatal clinic sites. The sites included two community health clinics, one county hospital prenatal clinic, and one private obstetrics and gynecology practice. Eligibility criteria for cohort entry included: (1) < 30 weeks pregnant, (2) $\ge$ 18 years of age, and (3) fluent in English or Spanish. Exclusion criteria for the study included: (1) multiple gestation; (2) having a physical, mental, or cognitive disability that would inhibit participation or the ability to provide consent; (3) current incarceration; or (4) HIV positive status. Informed consent and Health Insurance Portability and Accountability Act (HIPAA) authorization for medical record abstraction were obtained from each participant at time of study entry and the University of Southern California’s Institutional Review Board (IRB) approved all study aspects.

This study included participants who had a study-measured ultrasound conducted by a licensed sonographer during the third trimester visit ($\ge$ 28 weeks gestation). Of the 285 participants with a fetal ultrasound in the third trimester, one participant was missing data on prenatal ambient air pollution, and an additional three participants were missing information on race/ethnicity leaving a final sample size of 281 participants. Participants had similar sociodemographic characteristics to the overall MADRES cohort (Supplemental Table 1).

### Prenatal ambient air pollution measurements

Daily residential histories were assembled and geocoded for each participant using residential address and occupancy dates collected with residential history forms and prospective address confirmation data collected at every contact point. These capture all residential mobility or moves and form the basis of all geospatial exposure assignments. Daily ambient air pollutant concentrations of PM_2.5_, PM_10_, O_3_ (8-h maximum) and NO_2_, were then assigned using inverse-distance-squared weighted spatial interpolation from the United States Environmental Protection Agency (EPA) Air Quality System monitors. For PM_2.5_, PM_10_, and NO_2,_ 24-h daily averages were used, while the 8-h daily maximum was used for O_3._

Additionally, daily temperature in degrees Celsius was calculated as the average between the minimum and maximum temperature, which were obtained from a high-resolution (4 km × 4 km) gridded meteorological dataset [45]. Weekly averages were calculated from daily concentrations of pollutants and temperature from 12 weeks prior to conception until 32 weeks of gestation (44 total weeks). The weekly averages were computed with generally complete data (< 1% of daily concentrations were missing). Three participants had missing data on pollutants for certain weeks during pregnancy (4–22 weeks) due to inadequate quality of address geocode match or living abroad during a period of gestation. We chose to examine preconception windows up to 12 weeks prior to conception due to considering this as the biologically relevant window of exposure and previous literature using this time period for risk of gestational diabetes [36–38]. Exposures through 32 weeks gestation were chosen to correspond to the mean gestational age at ultrasound measurement across participants.

Overall pregnancy average exposures were calculated for each participant starting from date of conception until the date of the ultrasound scan (consisting of a mean of 224 $\pm$ 12 days).

### Fetal growth measurements

Five fetal growth outcomes were measured via transabdominal ultrasound. Outcomes included head circumference in millimeters (mm) defined as the length along the skull bone, biparietal diameter (mm) defined as the maximum diameter of a transverse section the fetal skull from the proximal parietal bone to the inner edge of the distal parietal bone, femur length (mm) defined as the length of the thigh bone, abdominal circumference (mm) which is measured at the widest part across the fetal liver and estimated fetal weight in grams (g) derived from the formula from Hadlock et al., which takes into account the above measurements [46–49].

This analysis used fetal biometry measurements from a single study-measured ultrasound scan (Mean: 32 $\pm$ 2 Range: 28–36 weeks gestation) conducted by certified, licensed sonographers at the third trimester study visit. A total of 65 scans were conducted from August 2016 to August 2018 by two sonographers at USC Keck Hospital with a Toshiba Aplio 500 machine. The sonographers conducted roughly equal numbers of scans (“sonographer A” 30 scans, “sonographer B” 35 scans). The remaining 216 scans were conducted by a single sonographer (“sonographer C”) at the MADRES clinic from August 2018 to March 2020 using a Philips CX-50 machine, with a convex 1-5 MHz transducer.

### Covariates

Covariates to include in multivariate models were identified a priori based on a review of the literature and visualized through Directed Acyclic Graphs (DAGs) [50]. Maternal and demographic covariates were included such as maternal age at time of study recruitment, maternal race and ethnicity (as a proxy for experiences of discrimination), maternal education level, annual household income, parity, pre-pregnancy body mass index (BMI), fetal sex, gestational age at time of ultrasound scan, temperature, season of ultrasound, recruitment site and ultrasound technician. We additionally considered personal smoking during pregnancy, but due to very few participants reporting any personal smoking during pregnancy (< 2.5%) it was not included as a covariate and was instead evaluated in a sensitivity analysis by removing participants that smoked.

Age, race/ethnicity, education, household income, birth order of the child, and smoking were self-reported via interviewer-administered questionnaires in English or Spanish. Pre-pregnancy BMI was computed using self-reported pre-pregnancy weight and study measured standing height using a stadiometer (Perspectives enterprises model PE-AIM-101). Sex of the fetus was abstracted from the electronic medical record (EMR) (98%) or came from the birth information form/proxy report from the mother (2%) and gestational age at time of ultrasound scan in weeks (including partial weeks by number of additional days) was calculated by subtracting the difference in number of weeks between the infant’s date of birth and the date of the ultrasound from the gestational age in weeks at time of birth. Gestational age at birth was calculated and standardized using a hierarchy of methods [51]. A first trimester (< 14 weeks gestation) ultrasound measurement of crown-rump length was considered highest quality and was used if available (59%). If missing, a second trimester (< 28 weeks gestation) ultrasound measurement of fetal biparietal diameter was used (27%). If measurements from an early ultrasound were unavailable, gestational age at birth was established from a physician’s best clinical estimate from the EMR (14%).

### Statistical analysis

Distributions of participant demographic and health characteristics were summarized using means and standard deviations for continuous variables and frequencies and percentages for categorical variables. Due to right skewed distributions, medians and interquartile ranges (IQR) were computed for each ambient air pollutant and we used Spearman correlations to assess the relationships between the air pollutants. Spaghetti plots were generated to visualize the change in pollutant exposure across the weeks of gestation for each participant. Means and standard deviations were calculated for each fetal growth outcome and Pearson correlations were computed to assess the relationships across the fetal biometry measurements due to meeting parametric assumptions.

We fitted DLMs [52] to estimate the time-varying associations between air pollutants at each week, from 12 weeks preconception to 32 weeks of gestation (44 total weeks), to determine critical windows of exposure. This approach allowed the effects of air pollution and temperature to be distributed across time with average weekly levels created using a “cross-basis” function which constrained correlation across weeks [52]. Within the DLMs, natural cubic splines with 2–8 degrees of freedom were tested while adjusting for covariates. Covariates were modeled as follows: age in years, pre-pregnancy BMI in kg/m^2^, gestational age at time of scan in weeks, week-specific average temperature in degrees Celsius, race/ethnicity (Hispanic, non-Hispanic Black, non-Hispanic White, non-Hispanic Other), education (high school diploma or less, some or completed college), household income (< $50,000, $$\ge$$ $50,000, or reported “Don’t Know”), birth order (first, second or more, missing data indicator), fetal sex (male or female), season at time of ultrasound scan (winter, fall, spring, or summer), technician, and recruitment site. The model with the lowest Akaike information criteria AIC was chosen and knots were placed at weeks 2 and 20 of gestation. The regression slope for weekly air pollution exposure was scaled to an IQR increase for the pollutant. A sensitive window was defined as week(s) when the 95% confidence intervals (CI) did not include zero. Effect modification by fetal sex was evaluated through stratified models.

Sensitivity analyses were conducted to further evaluate robustness of results by first removing participants who reported any smoking during pregnancy (< 2.5%) and then separately by adjusting for high-risk pregnancies captured by gestational or chronic diabetes and hypertension reported on the EMR (49%). Lastly, we assessed results when additionally adjusting for total gestational weight-gained in pregnancy (kg) and physical activity during pregnancy (MET-h-week^−1^). Total gestational weight gain was quantified as the difference between the pregnant individual’s weight measured within two weeks before giving birth and their weight prior to pregnancy (Mean: 10.8 $\pm$ 7.4 kg). Pre-pregnancy weight was self-reported through interviewer-administered questionnaires during pregnancy (95.7%). If missing, the first weight measured during the pregnancy was obtained from the EMR (4.3%). Physical activity was captured using the total activity score from the pregnancy physical activity questionnaire (PPAQ) [53] collected in the third trimester of pregnancy (Mean: 282.8 $\pm$ 122.3 MET-h-week^−1^). DLMs were fit using the dnlm package within R [52]. Although this package allows for non-linear modeling approaches, we only considered linear air pollution effects at a given lag, which was confirmed appropriate by spline tests using generalized additive models (GAM) with overall prenatal air pollution and each growth outcome (*p* > 0.05).

To compare results within MADRES to more traditionally used linear regression model results, a secondary analysis was conducted using the overall pregnancy average concentration of each pollutant from date of conception until date of the ultrasound scan as the exposure with each of the fetal biometry outcomes. Linear regression models were adjusted for the same covariates. Beta estimates for the exposure were standardized to the pollutant’s IQR in order to aid with interpretation. Additionally, multipollutant models were then conducted to assess robustness of single pollutant results. Influential points were assessed through Jackknife residuals and Cook’s D and all models met the assumptions of linear regression. Data cleaning, management, and linear regression models were conducted in SAS Version 9.4 and DLM analyses were conducted with 4.04. Version of R. All analyses were conducted with two-sided hypotheses and an alpha level of 0.05.

## Results

### Participant characteristics

Participants were on average 29 $$\pm$$ 6 years of age, primarily overweight prior to pregnancy (Mean BMI = 29 $$\pm$$ 7), predominately Hispanic (82%), nearly half had family incomes of less than $50,000 per year (44%), and a majority of participants had a high school diploma or less education (58%). There were slightly more female fetuses (52%), and the majority of participants were pregnant with at least their second child (69%). Participant characteristics are shown in Table 1.

**Table 1:** Participant Characteristics (*N* = 281)

Characteristic	Mean (SD) or N (%)
**Participant**	
Race/Ethnicity	
Hispanic	230 (81.9%)
Non-Hispanic Black	36 (12.8%)
Non-Hispanic White	8 (2.8%)
Non-Hispanic Other	7 (2.5%)
Annual Household Income	
< $50,000	123 (43.8%)
$$\ge$$$50,000	52 (18.5%)
Reported “Don’t Know”	106 (37.7%)
Education	
HS Diploma or Less	164 (58.4%)
Some or Completed College	117 (41.6%)
Age (years)	28.5 (6.1)
Pre-Pregnancy BMI (kg/m^2^)	29.2 (6.7)
Any Prenatal Smoking	7 (2.5%)
**Fetus**	
Sex	
Female	146 (52.0%)
Gestational Age at Ultrasound (weeks)	31.8 (1.7)
Birth Order	
First	80 (28.5%)
Second or more	195 (69.4%)
Unknown	6 (2.1%)

### Ambient air pollution

Median and IQR of averaged PM_2.5_, PM_10_, NO_2_, and O_3_ concentrations from date of conception to date of ultrasound and median and IQR of averaged weekly (12 weeks preconception to 32 weeks of gestation) concentrations are shown in Table 2. Overall pregnancy average 8-h maximum O_3_ concentrations were inversely correlated with 24-h NO_2_ (*R* = -0.61, *p* < 0.001) and 24-h PM_2.5_ (*R* = -0.16, *p* = 0.007), while positively correlated with 24-h PM_10_ (*R* = 0.22, *p* = 0.0002)_._ NO_2_ was positively correlated with both PM_2.5_ (*R* = 0.46, *p* < 0.001) and PM_10_ (*R* = 0.20, *p* = 0.001). PM_2.5_ and PM_10_ were positively correlated with one another (*R* = 0.64, *p* < 0.001). Plots of weekly pollutant correlations across gestation (Week 1 to Week 32) are shown in Supplemental Fig. 1.

**Table 2:** Distributions of Pregnancy Average and Weekly Average Ambient Air Pollutants

Pollutant	Pregnancy Average Median (IQR)	Weekly Average Median (IQR)
PM_2.5_ ($\mu$ g/m^3^)	11.7 (2.2)	11.7 (1.9)
PM_10_ ($\mu$ g/m^3^)	27.9 (7.1)	29.5 (6.5)
NO_2_ (ppb)	15.5 (5.6)	15.3 (5.6)
O_3_ (ppb)	42.3 (5.7)	42.9 (5.6)

Note: PM_2.5_, PM_10,_ and NO_2_ are from 24-h estimates, O_3_ represents the 8-h maximum

Pregnancy average: from date of conception to date of ultrasound scan

Weekly average: from 12 weeks preconception to 32 weeks of gestation

### Fetal growth outcomes

Fetal growth was measured via ultrasound at a mean of 32 weeks $\pm$ 2 gestation and the majority of scans were conducted in Fall or Winter (32% Fall, 31% Winter, 19% Spring, 18% Summer). Fetal biometry outcomes were normally distributed (Shapiro Wilk *p* values > 0.05) with the exception of estimated fetal weight, which was slightly left skewed. Means and standard deviations are shown in Table 3. All outcomes were significantly positively correlated with one another (Pearson’s R 0.75 to 0.97, *p* < 0.0001). Fetal growth outcomes had weak to moderate positive significant correlations with infant birth weight. Correlations are shown in Supplemental Fig. 2.

**Table 3:** Descriptive Statistics of Fetal Growth Outcomes (Mean: 32 $\pm$ 2 weeks of gestation)

Fetal Growth Outcome	N	Mean (SD)
Estimated Fetal Weight (g)	281	1957.0 (376.9)
Head Circumference (mm)	281	297.7 (14.1)
Abdominal Circumference (mm)	281	282.7 (19.9)
Biparietal Diameter (mm)	281	80.6 (4.3)
Femur Length (mm)	279	61.3 (3.4)

### Prenatal air pollution exposure and fetal growth

**Fig. 1:**
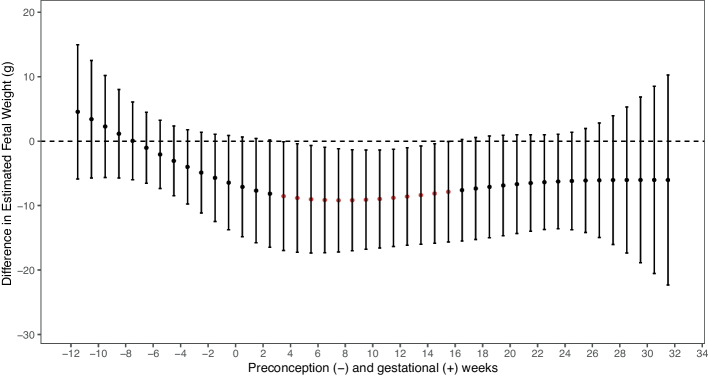
DLM Results Relating Weekly PM_2.5_ Exposure to Estimated Fetal Weight per IQR Note: red estimate *p* < 0.05; IQR = 6 $\mathrm{\mu g}/\mathrm{m}$^3^ Adjusted for maternal age at time of study recruitment, maternal race and ethnicity, maternal education level, household income, parity, pre-pregnancy body mass index (BMI), sex of the fetus, gestational age at time of ultrasound scan, lag specific average temperature, season of ultrasound, ultrasound technician, and recruitment site

**Fig. 2:**
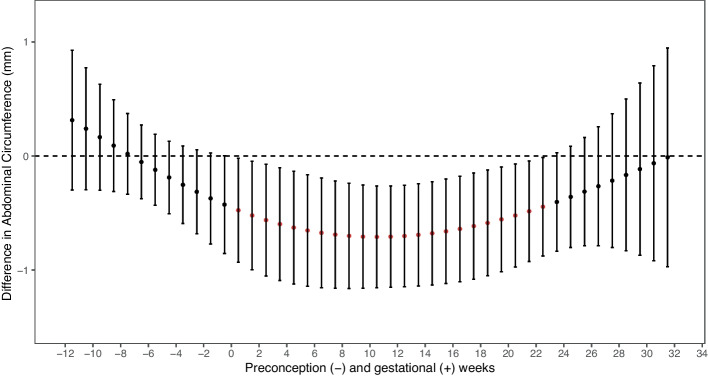
DLM Results Relating Weekly PM_2.5_ Exposure to Fetal Abdominal Circumference per IQR Notes: red estimate *p* < 0.05; IQR = 6 $\mathrm{\mu g}/\mathrm{m}$^3^ Adjusted for maternal age at time of study recruitment, maternal race and ethnicity, maternal education level, household income, parity, pre-pregnancy body mass index (BMI), sex of the fetus, gestational age at time of ultrasound scan, lag specific average temperature, season of ultrasound, ultrasound technician, and recruitment site

**Fig. 3:**
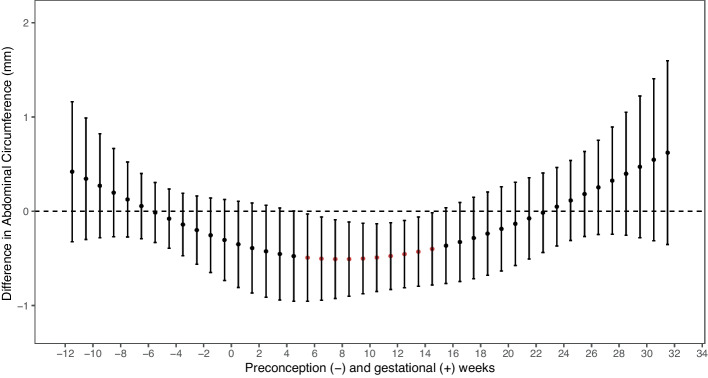
DLM Results Relating Weekly PM_10_ Exposure to Fetal Abdominal Circumference (mm) per IQR Notes: red estimate *p* < 0.05; IQR = 13 $\mathrm{\mu g}/\mathrm{m}$^3 ^Adjusted for maternal age at time of study recruitment, maternal race and ethnicity, maternal education level, household income, parity, pre-pregnancy body mass index (BMI), sex of the fetus, gestational age at time of ultrasound scan, lag specific average temperature, season of ultrasound, ultrasound technician, and recruitment site

We investigated weekly lagged exposure from 12 weeks preconception to 32 weeks of gestation. After adjustment for covariates, we found significant windows of exposure in which PM_2.5_ and PM_10_ had inverse associations with fetal abdominal circumference and PM_2.5_ with estimated fetal weight (Figs. 1 and 2). We observed a significant sensitive window of PM_2.5_ exposure (per IQR increase of 6 $\mathrm{\mu g}/\mathrm{m}$ ^3^) between weeks 4–16 for lower estimated fetal weight $(\beta$_averaged4-16_ = -8.7 g; 95% CI -16.7, -0.8). The strongest effect size was at week 8 of pregnancy ($\beta$= -9.3 g; 95% CI -17.3, -1.2). Exposure to PM_2.5_ during gestational weeks 1–23 was also significantly associated with smaller fetal abdominal circumference ($\beta$_averaged1-23_ = -0.6 mm; 95% CI -1.1, -0.2) with the strongest effect size at week 11 of pregnancy ($\beta$= -0.7 mm; 95% CI -1.2, -0.3). Similar associations for PM_10_ were observed with abdominal circumference (Fig. 3). Prenatal exposure to PM_10_ (per IQR increase of 13 $\mathrm{\mu g}/\mathrm{m}$ ^3^) between weeks 6–15 of pregnancy was significantly associated with smaller third trimester fetal abdominal circumference ($\beta$_averaged6-15 =_ -0.4 mm; 95% CI -0.8, -0.1) with the strongest effect size at week 9 of pregnancy ($\beta$= -0.5 mm; 95% CI -0.9, -0.1). No other ambient air pollutants or fetal growth outcomes showed significant weeks of exposure through DLMs.

In stratified models, we found different patterns of associations for PM_10_ exposure and abdominal circumference by sex. Critical windows of exposure were identified among female fetuses (*N* = 146) between weeks 12–14, while none were observed among male fetuses (*N* = 135) (Supplemental Fig. 3). Among female fetuses, a one IQR increase in PM_10_ was associated with $\beta$_averaged12-14_ = -0.5 mm (95% CI -1.1, -0.1) smaller fetal abdominal circumference. No other significant windows were identified in stratified models. Overall results were consistent when we removed participants with any reported smoking during pregnancy (*N* = 7) (Supplemental Fig. 4), when we additionally adjusted for maternal health complications (chronic and gestational diabetes/hypertension) (Supplemental Fig. 5), and when we additionally adjusted for total gestational weight gain and physical activity in pregnancy (Supplemental Fig. 6).

Looking at pregnancy-wide exposures and fetal growth using fully adjusted linear regression models, our findings were consistent (Table 4). Per one IQR (IQR = 2.2 g$\mu$/m^3^) increase in prenatal average PM_2.5_ exposure, fetal abdominal circumference decreased by -3.8 mm (95% CI -7.1, -0.4). Additionally, estimated fetal weight decreased by -57.5 g (95% CI -114.5, -0.4). Results remained statistically significant in multipollutant models that additionally adjusted for O_3_ (PM_2.5_ and estimated fetal weight $\beta$ = -64.7 g; 95% CI -127.7, -1.6 and PM_2.5_ and abdominal circumference $\beta$= -4.2 mm; 95% CI -7.9, -0.5). There were no other significant associations between prenatal averaged ambient air pollutants and fetal growth, although PM_10_ exposure and fetal abdominal circumference suggested an inverse trend ($\beta$ = -1.8 mm per IQR; 95% CI -4.9, 1.3).

**Table 4:** Linear Regression Results for Prenatal Average Air Pollution Exposure and Fetal Growth

	EFW (g)^a^	HC (mm)^a^	AC (mm)^a^	BPD (mm)^a^	FL (mm)^a^
	$\upbeta$(95% CI)	$\upbeta$(95% CI)	$\upbeta$(95% CI)	$\upbeta$(95% CI)	$\upbeta$(95% CI)
NO_2_ (ppb)	-35.6 (-98.7, 27.5)	-1.2 (-4.0, 1.6)	-2.6 (-6.3, 1.2)	0.001 (-0.9, 0.9)	0.1 (-0.6, 0.9)
O_3_ (ppb)	13.6 (-38.1, 65.2)	-0.7 (-3.0, 1.6)	0.8 (-2.3, 3.8)	0.2 (-0.5, 1.0)	0.2 (-0.4, 0.8)
PM_2.5_ ($\mu$ g/m^3^)	-57.5 (-114.5, -0.4)*	-1.0 (-3.5, 1.6)	-3.8 (-7.1, -0.4)*	-0.4 (-1.2, 0.4)	-0.3 (-1.1, 0.3)
PM_10_ ($\mu$ g/m^3^)	-22.4 (-75.5, 30.7)	0.3 (-2.0, 2.7)	-1.8 (-4.9, 1.3)	-0.2 (-0.9, 0.6)	0.3 (-0.5, 0.7)

Beta estimates are standardized to the IQR of the pregnancy average ambient air pollutant NO_2_ = 5.6 ppb; O_3_ = 5.7 ppb; PM_2.5_ = 2.2 g$\mu$/m^3^ PM_10_ = 7.1 g$\mu$/m^3^

*EFW* Estimated Fetal Weight, *HC* Head Circumference, *AC* Abdominal Circumference, *BPD* Biparietal Diameter, *FL* Femur Length

^a^Adjusted for maternal age at time of study recruitment, maternal race and ethnicity, maternal education level, household income, parity, pre-pregnancy body mass index (BMI), sex of the fetus, gestational age at time of ultrasound scan, average temperature, season of ultrasound, ultrasound technician, and recruitment site

^*^p < 0.05;

## Discussion

In this study of primarily low-income Hispanic participants residing in urban Los Angeles, we identified critical windows of prenatal exposure to PM_2.5_ that were significantly associated with lower fetal growth. Exposure to PM_2.5_ during weeks 4 to 16 of pregnancy was associated with lower estimated fetal weight and exposure from weeks 1 to 23 of pregnancy was associated with smaller fetal abdominal circumference. The strongest effect sizes were observed at week 8 and 11, respectively. We additionally found that prenatal PM_10_ exposure from weeks 6 to 15 of pregnancy was associated with smaller fetal abdominal circumference, with the strongest effect size at week 9.

Our results are consistent with four studies that have assessed the impacts of prenatal PM_2.5_ exposure across averaged time windows on in utero fetal growth [34, 38–40]. Leung et al. assessed spatiotemporal modeled PM_2.5_ exposure and fetal growth among predominately non-Hispanic white participants in Eastern Massachusetts [39]. They found average PM_2.5_ from conception through 16 weeks of gestation was associated with reduced abdominal circumference measured at both < 24 weeks and $\ge$ 24 weeks. Lin et al., assessed average ambient PM_2.5_ exposure from date of last menstrual period to date of ultrasound on estimated fetal weight in a study in Beijing, China and found reduced fetal weight with increasing PM_2.5_ exposure [40]. Cao et al. found average prenatal ambient PM_2.5_ from conception to one week prior to ultrasound was associated with reduced fetal abdominal circumference and estimated fetal weight in participants in Shanghai, China [38]. Clemens et al. found non-significant inverse associations between annual average PM_2.5_ concentrations with abdominal circumference in a study in Scotland [34]. Consistent with our results, two previous studies have found averaged prenatal PM_10_ exposure to have significant inverse effects on fetal abdominal circumference [35, 36]. Of the studies that have assessed PM_10_ on fetal abdominal circumference [31, 34–37], none stratified results by fetal sex except Clemens et al. within sensitivity analyses, and results were not shown [34]. Previous research has suggested female fetuses are more susceptible to exposures late in the first trimester and during the second trimester, possibly indicated by more female fetus pregnancy losses compared to male fetuses during this time period [54]. This would overlap with the significant weeks identified in our study.

Normal fetal growth across gestation is a critical component of a healthy pregnancy. Intrauterine growth restriction and slow in utero growth can influence the long-term health of the child including increased risk for type 2 diabetes mellitus, coronary heart disease, and hypertension [25–27]. Markers of abnormal gestational growth have been associated with later life outcomes. For example, reduced fetal head circumference has been associated with reduced childhood intelligence quotient (IQ) [55] and smaller fetal abdominal circumference with childhood allergy outcomes [56] and BMI [57]. Suggested biological mechanisms for how prenatal air pollution influences gestation include impacts on the placenta, which is a susceptible target of environmental insults, and disruption of placental function can lead to altered fetal growth [21]. A recent review indicated that prenatal air pollution exposure is associated with both nitrosative stress and epigenetic changes in the placenta [24]. Specifically, PM has been associated with oxidative stress leading to placental inflammation and eventually impaired transplacental oxygen and nutrient delivery to the fetus by triggered hemodynamic responses, ultimately impacting fetal growth [58].

This current study has several notable strengths. MADRES is a well-characterized longitudinal cohort study. The cohort represents a structurally marginalized population who have traditionally been excluded from research and have higher exposure to environmental contaminants due to structural inequities based on race and ethnicity [7, 8]. Within the United States, pregnant persons of color, specifically Black and Hispanic populations, have higher rates of preterm birth and low birth weight when compared to their non-Hispanic white counterparts [10]. Communities of color are also disproportionally burdened by environmental health hazards including air pollution [6, 59]. Previous research has suggested that policies that focus on reducing air pollution exposure have the potential to decrease the Black-white disparities of preterm birth [60].

Another strength of the study was the detailed residential histories, which allowed for finely time-resolved estimates of exposure and accounted for housing relocations during the preconception and gestational time periods. We used these daily estimates to not only create overall averages, which is currently the primary method of exposure modeling within these associations in the literature, but also to create weekly averages to include in DLMs. A key strength of the DLM framework is that it utilizes the data from all weeks concurrently, and assumes that the association varies smoothly as a function of time, while also adjusting for the exposure levels at the different weeks included [61]. This allowed us to identify critical windows of exposure while also not increasing the likelihood for a Type 1 error.

Including preconception air pollution exposure in the modeling framework is an additional strength as it is a currently understudied potential critical period of susceptibility for fetal growth effects. Although our study examined exposure to air pollution three months prior to conception, no critical windows of exposure on fetal growth were identified during this preconception period. Emerging literature has shown that preconception air pollution exposure influences pregnancy outcomes, notably gestational diabetes mellitus [41–43, 62]. The influence of preconception exposure to air pollution on fetal growth may be smaller than that of exposure during pregnancy, thus indicating more statistical power may be needed through larger sample sizes. There are currently no other studies that have examined the effects of preconception air pollution on in utero fetal growth, and future studies should explore this further when investigating critical windows of exposure.

As in all observational studies, the current study also has limitations to address. Due to the urban environment of Los Angeles, our results may not be generalizable to other regions with a different mix of air pollution sources or those with higher or lower levels. Additionally, although medical record data were available for chronic and gestational health outcomes related to diabetes and hypertension, measurement error in confounders is possible. We also were unable to assess the potential modifying effects of prenatal smoking, due to very few participants reporting any smoking during pregnancy, although this likely eliminated any confounding effect from personal smoking during gestation. It is a potential limitation that we did not have information to confirm that participants were also primarily non-smokers prior to pregnancy. Our study also only included participants that had ultrasounds taken in the third trimester, which would inherently exclude pregnancies that had ended in early miscarriages. This may also be why we did not see critical windows of exposure during preconception. However, results from a recent simulation study have shown live birth bias may have a negative bias which would mean results would likely be stronger than what was observed within this study if present [63].

## Conclusions

Overall, this study provides compelling evidence that early to mid-pregnancy exposure to ambient air pollution, particularly PM_2.5_, influences fetal growth in utero*.* Our results add to the growing literature that exposure to ambient air pollution during the susceptible prenatal period influences fetal health and development, which ultimately may affect health later in life. Air pollution is a modifiable environmental exposure and continued efforts to reduce air pollution are needed to protect vulnerable populations.

## Supplementary Information

**Additional file 1: Supplemental Table 1.** Demographics of 863 participants within the MADRES Study. **Supplemental Figure 1.** Spearman Correlations of Pollutants Weeks 1-32 of Gestation. **Supplemental Figure 2.** Pearson Correlations of Fetal Growth Outcomes With Infant Birth Weight. **Supplemental Figure 3. **DLM Model Results for PM_10_ and Fetal Abdominal Circumference Stratified by Fetal Sex. **Supplemental Figure 4.** Results of DLM Models After Additionally Adjusting for Chronic/Gestational Diabetes and Hypertension. **Supplemental Figure 5.** Results of DLM Models After Removing Mothers Who Reported Any Smoking (*N*=7). **Supplemental Figure 6.** Results of DLM Models After Additionally Adjusting for Gestational Weight Gain and Physical Activity in Pregnancy

## Data Availability

The datasets generated and/or analyzed during the current study are not publicly available due to containing information that could compromise the privacy of research participants but are available from the corresponding author on reasonable request.

## Abbreviations

PM_2.5_: Particulate Matter with an aerodynamic diameter of 2.5 $\upmu$ m or less
PM_10_: Particulate Matter with an aerodynamic diameter of 10 $\upmu$ m or less
NO_2_: Nitrogen
O_3_: Ozone
DAG: Directed Acyclic Graph
PPAQ: Pregnancy Physical Activity Questionnaire
EMR: Electronic Medical Record
IQR: Interquartile Range
DLM: Distributed Lag Model
GAM: Generalized Additive Model
