# Supplementary material for: Identifying pre-conception and pre-natal periods in which ambient air pollution exposure affects fetal growth in the predominately Hispanic MADRES cohort
Source: Environ Health. 2022 Nov 26;21:115. doi: 10.1186/s12940-022-00925-0 (PMC9701016; doi:10.1186/s12940-022-00925-0)
Supplement: Supplementary file 1 — Additional file 1: Supplemental Table 1. Demographics of 863 participants within the MADRES Study. Supplemental Figure 1. Spearman Correlations of Pollutants Weeks 1-32 of Gestation. Supplemental Figure 2. Pearson Correlations of Fetal Growth Outcomes With Infant Birth Weight. Supplemental Figure 3. DLM Model Results for PM10 and Fetal Abdominal Circumference Stratified by Fetal Sex. Supplemental Figure 4. Results of DLM Models After Additionally Adjusting for Chronic/Gestational Diabetes and Hypertension. Supplemental Figure 5. Results of DLM Models After Removing Mothers Who Reported Any Smoking (N=7). Supplemental Figure 6. Results of DLM Models After Additionally Adjusting for Gestational Weight Gain and Physical Activity in Pregnancy [file 12940_2022_925_MOESM1_ESM.docx]

Supplemental Material

**Supplemental Table 1.** **Demographics of 863 participants within the MADRES Study**

| Characteristic | Mean (SD) / N (%) |
| --- | --- |
| Race/Ethnicity (N=811)  Hispanic  Non-Hispanic Black  Non-Hispanic White  Non-Hispanic Other | 637 (78.5%)  102 (12.6%)  44 (5.4%)  28 (3.5%) |
| Household Income (N=804)  <$50,000  >$50,000  Reported “Don’t Know” | 454 (56.5%)  78 (9.7%)  272 (33.8%) |
| Education (N=787)  High School Diploma or Less  Some College/Technical School | 459 (58.3%)  328 (41.7%) |
| Age at recruitment (years) | 28.3 (6.1) |
| Pre-pregnancy BMI (N=768) | 28.8 (6.8) |
| Any Prenatal Smoking | 17 (2.0%) |

**Supplemental Figure 1. Spearman Correlations of Pollutants Weeks 1-32 of Gestation**


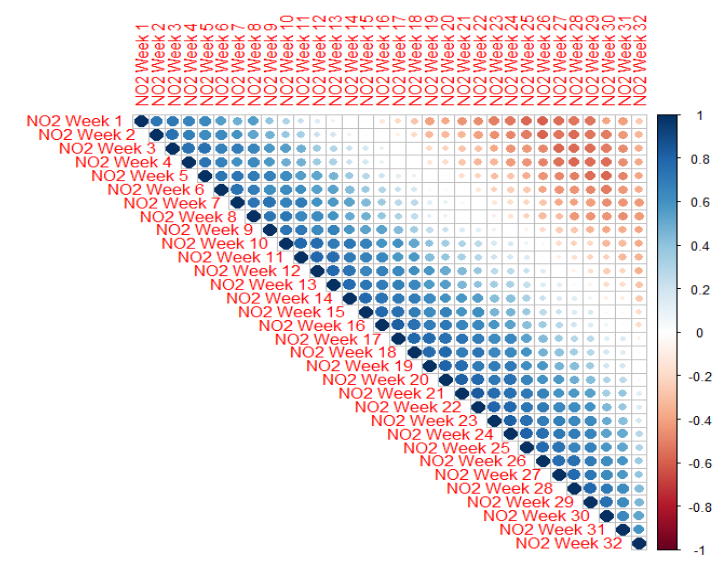

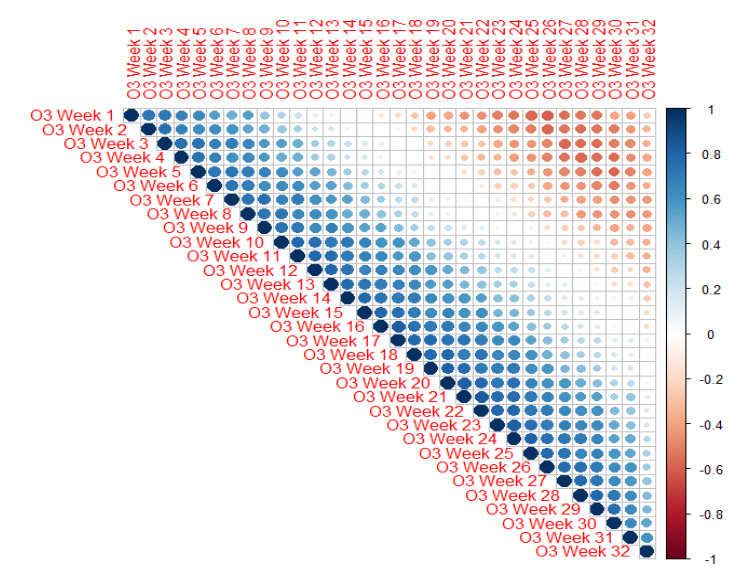

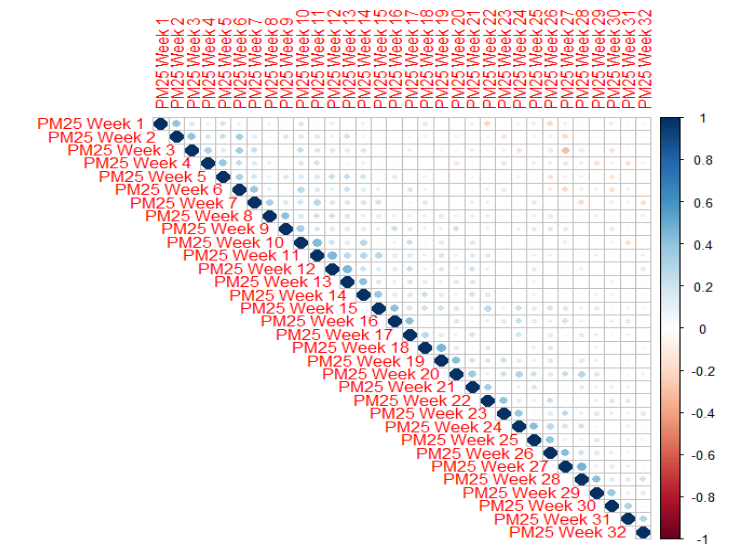

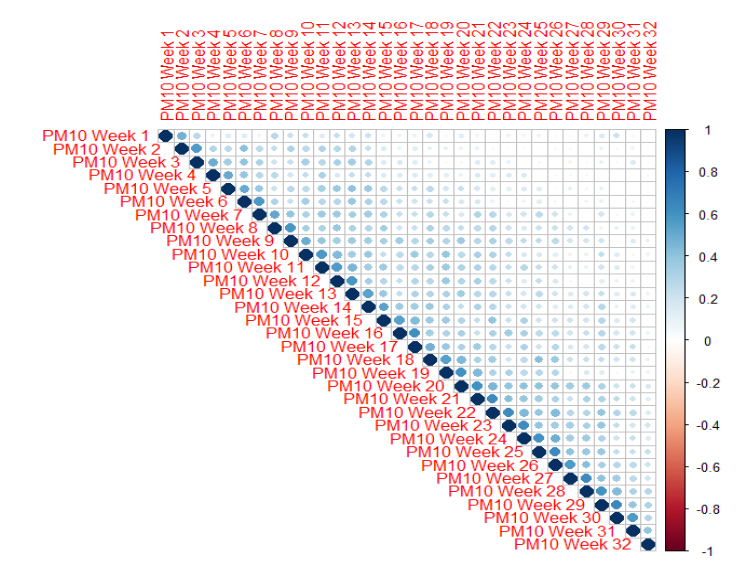


**Supplemental Figure 2. Pearson Correlations of Fetal Growth Outcomes With Infant Birth Weight**


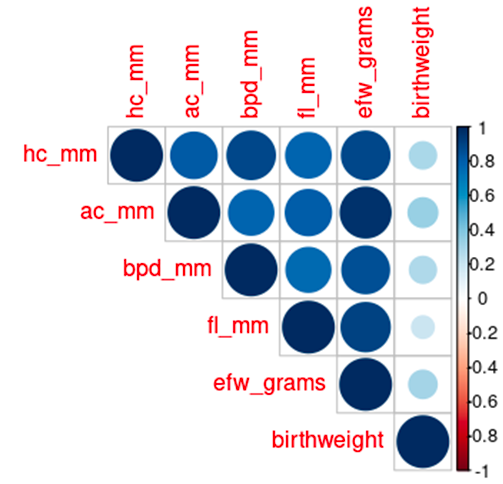


Note: HC= Head Circumference, AC=abdominal circumference, BPD=Biparietal Diameter, EFW=Estimated Fetal Weight

**Supplemental Figure 3. DLM Model Results for PM_10_ and Fetal Abdominal Circumference Stratified by Fetal Sex**


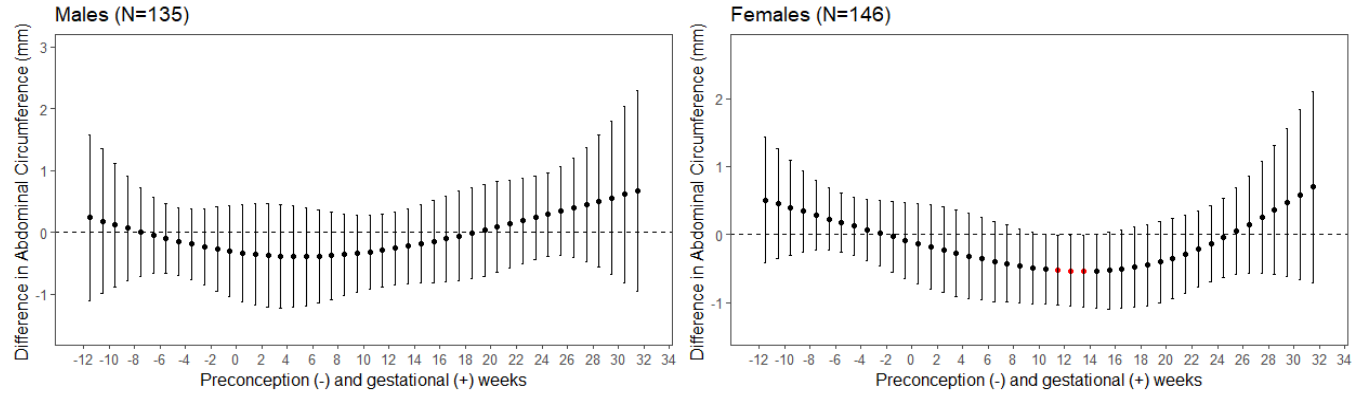

Adjusted for maternal age at time of study recruitment, maternal race and ethnicity, maternal education level, household income, parity, pre-pregnancy body mass index (BMI), gestational age at time of ultrasound scan, lag specific average temperature, season of ultrasound, ultrasound technician, and recruitment site. Note: red estimate p<0.05; IQR=13$\mu g/m$^3^

**Supplemental Figure 4. Results of DLM Models After Additionally Adjusting for Chronic/Gestational Diabetes and Hypertension**


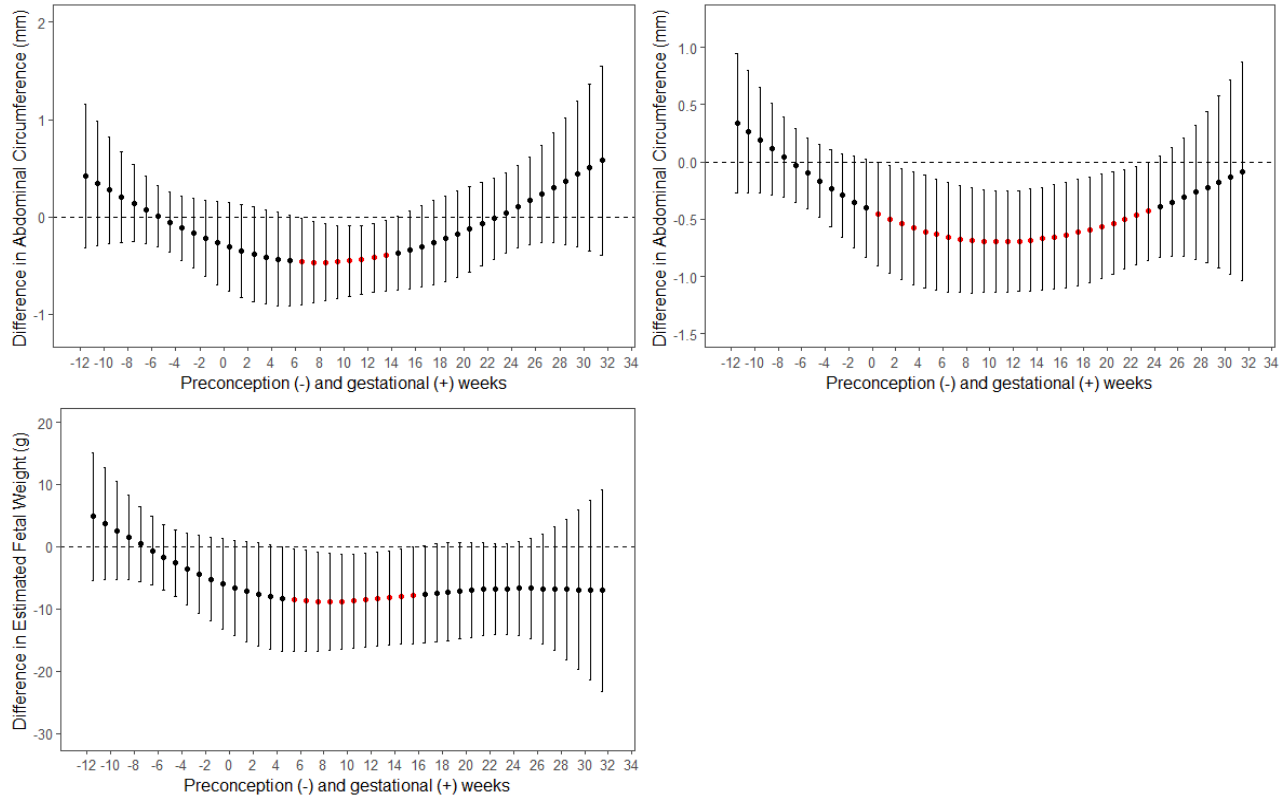


PM_10_

PM_2.5_

PM_2.5_

Adjusted for maternal age at time of study recruitment, maternal race and ethnicity, maternal education level, household income, parity, pre-pregnancy body mass index (BMI), sex of the fetus, gestational age at time of ultrasound scan, lag specific average temperature, season of ultrasound, ultrasound technician, recruitment site, and maternal health complications (chronic/gestational diabetes and hypertension). Note: red estimate p<0.05; PM_2.5_ IQR=6$\mu g/m$^3^ ; PM_10_ IQR=13$\mu g/m$^3^

**Supplemental Figure 5. Results of DLM Models After Removing Mothers Who Reported Any Smoking (N=7)**


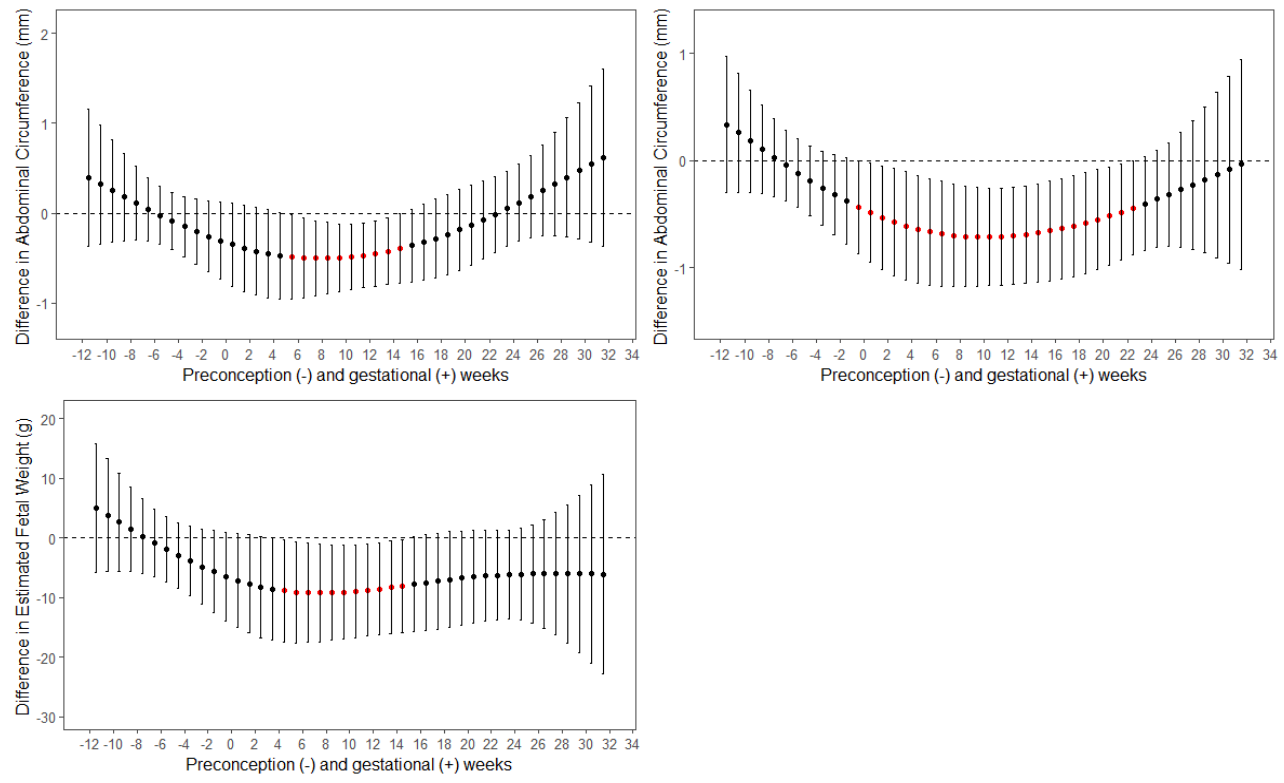


PM_10_

PM_2.5_

PM_2.5_

PM_10_

PM_2.5_

PM_2.5_

Adjusted for maternal age at time of study recruitment, maternal race and ethnicity, maternal education level, household income, parity, pre-pregnancy body mass index (BMI), sex of the fetus, gestational age at time of ultrasound scan, lag specific average temperature, season of ultrasound, ultrasound technician, and recruitment site. Note: red estimate p<0.05; PM_2.5_ IQR=6$\mu g/m$^3^ ; PM_10_ IQR=13$\mu g/m$^3^

**Supplemental Figure 6. Results of DLM Models After Additionally Adjusting for Gestational Weight Gain and Physical Activity in Pregnancy**


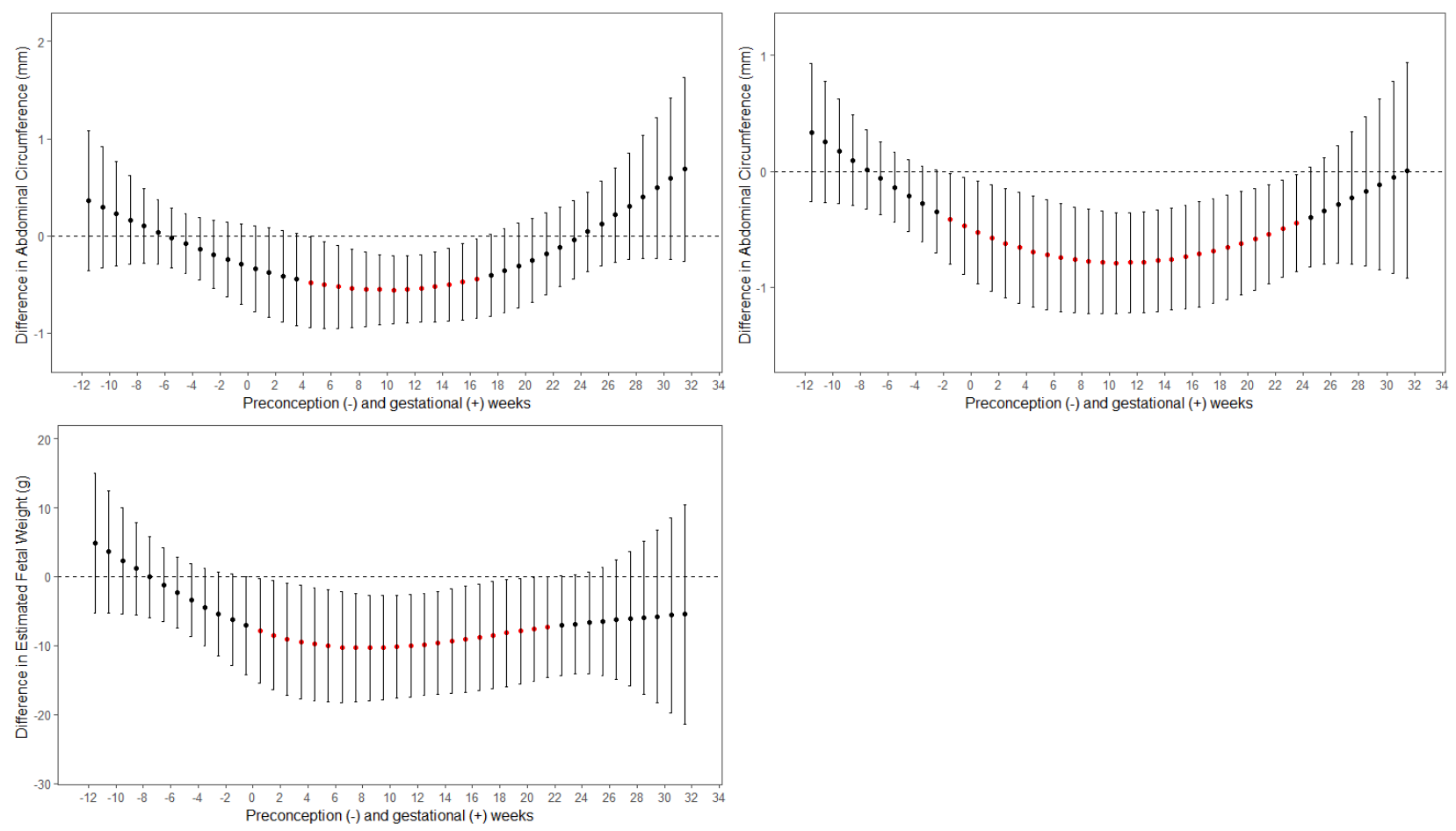


PM_10_

PM_2.5_

PM_2.5_

Adjusted for maternal age at time of study recruitment, maternal race and ethnicity, maternal education level, household income, parity, pre-pregnancy body mass index (BMI), sex of the fetus, gestational age at time of ultrasound scan, lag specific average temperature, season of ultrasound, ultrasound technician, recruitment site, total gestational weight gain, and physical activity. Note: red estimate p<0.05; PM_2.5_ IQR=6$\mu g/m$^3^ ; PM_10_ IQR=13$\mu g/m$^3^
